# Effectiveness and Safety of Intravenous Iron Therapy in Outpatient Obstetrical Clinic for Treatment of Iron‐Deficiency Anemia During Pregnancy

**DOI:** 10.1002/pmf2.70067

**Published:** 2025-08-22

**Authors:** Ellen M. Murrin, Olivia S. LeBeau, Lillian Singer, Mark A. Kassab, Peyton Kalan, Julie Costanzo, Scott A Sullivan, George L. Maxwell, George R. Saade, Antonio F. Saad

**Affiliations:** ^1^ Department of Obstetrics and Gynecology, Division of Maternal‐Fetal Medicine Inova Fairfax Hospital Falls Church Virginia USA; ^2^ Division of Maternal‐Fetal Medicine, Department of Obstetrics & Gynecology Eastern Virginia Medical School Norfolk Virginia USA

**Keywords:** antenatal care, hemoglobin levels, intravenous iron therapy, iron‐deficiency anemia, maternal health, outpatient treatment, pregnancy

## Abstract

**Background:**

Iron‐deficiency anemia (IDA) affects over one‐third of pregnant patients globally, contributing to severe maternal morbidity and adverse neonatal outcomes. Oral iron supplementation, while cost‐effective, is limited by poor absorption, significant gastrointestinal side effects, and poor adherence. The use of intravenous (IV) iron addresses many of these concerns, including eliminating GI side effects and the need for daily therapy. However, administration of IV iron in pregnant patients is typically restricted to specialized infusion centers or hospitals due to concerns about acute side effects, cost, resource utilization, the need for fetal heart rate monitoring, and extended infusion times.

**Methods:**

This descriptive study evaluated the implementation, safety, and feasibility of outpatient IV iron infusions for pregnant patients with IDA, with maternal hemoglobin response and outcomes reported as secondary findings. Patients received a single or series of doses of IV iron spaced weekly, with hemoglobin levels monitored before and after treatment. No fetal heart tracing was performed. Antepartum patients treated with IV iron at our outpatient obstetric clinic from 2017 to 2024 were identified retrospectively, and their charts were reviewed.

**Results:**

A total of 417 pregnant patients received IV iron (87.3% iron sucrose; 12.7% Ferric Carboxymaltose); 65.9% received ≥600 mg total dose (2–3 dose protocol), while 34.1% received lower cumulative doses (1–2 infusions). Therapy was well‐tolerated with no anaphylactic reactions reported. Side effects were experienced in only 11.5% of patients and were relatively minor, with dizziness (6.2%), headache (1.9%), and hypotension (1.7%) being the most common. Adverse maternal outcomes (PPH, ICU admission, blood transfusions, and re‐admissions) were low, particularly in patients who received the last infusion more than 10 days before delivery. Neonatal outcomes included a 7.4% NICU admission rate and a 3.5% rate of infants weighing less than 2500 g. Mean hemoglobin increased by 1.5 g/dL, consistent with prior studies.

**Conclusion:**

This study demonstrates that IV iron therapy can be safely given in obstetrical clinics without significant maternal or fetal safety concerns. This obviates the need for more resource‐intensive and costly settings such as infusion or hospital‐based centers.

## 1 INTRODUCTION

Iron deficiency anemia (IDA) is a prevalent condition affecting more than one‐third of pregnant patients globally [1, 2, 3] and is responsible for over 50% of antepartum anemia cases [2]. IDA is associated with severe maternal morbidity and adverse neonatal outcomes, including preterm birth, low birth weight, and increased perinatal mortality [2, 3]. Infants born to women with IDA are themselves at greater risk of iron deficiency, which in turn leads to delays in language and motor development [2, 3]. The standard management of IDA involves antenatal iron supplementation, typically administered orally due to its cost‐effectiveness in the outpatient setting [2, 4]. However, oral iron therapy is often limited by poor absorption, which is hepcidin‐dependent [5], significant gastrointestinal side effects, and poor patient adherence, with studies reporting up to a 50% compliance rate [6, 7, 8].

Intravenous (IV) iron therapy presents an alternative to oral supplementation, offering significant benefits such as consolidated treatment, obviating the need for absorption, and fewer gastrointestinal side effects [7].

The increased speed of repletion with IV iron makes it a particularly appealing option in pregnancy. Women are often diagnosed based on their third‐trimester labs, and oral iron can take up to six months to normalize ferritin [7]. The use of IV iron in the antepartum setting has decreased patients' need for blood transfusions at the time of delivery [9]. A recent meta‐analysis of ten RCTs demonstrates that IV iron is superior to oral iron for managing anemia during pregnancy [10]. IV iron more effectively achieves target hemoglobin (Hgb) levels, increases Hgb more rapidly, and is associated with fewer adverse reactions [10].

Despite these advantages, IV iron administration is often limited due to outdated beliefs regarding potential side effects, such as anaphylaxis. This practice necessitates the use of specialized infusion centers or hospital settings, primarily due to elevated costs and the requirement for resource utilization, including non‐stress tests (NSTs) and extended visit durations of up to 6–8 h [7, 11, 12, 13]. However, this practice is not supported by strong evidence. Clinical trials of IV iron in pregnancy vary in their approach: some do not describe fetal monitoring procedures at all, while others report pre‐ and post‐infusion fetal heart monitoring without demonstrating a clear need for such practice [14]. Recent guidelines do not recommend fetal heart monitoring and endorse consolidating care to a single infusion visit, further reducing the burden on resources and patients [15].

To address these challenges, we evaluated the safety and efficacy of IV iron therapy administered in an outpatient obstetric clinic while assessing maternal and fetal outcomes. This study suggests the potential for a wider implementation of outpatient IV iron therapy. It supports its role as a viable alternative to traditional hospital‐based administration or resource‐intense specialized infusion centers for treating IDA in pregnancy.

## 2 METHODS

We conducted a retrospective chart review study of outpatient IV iron therapy for the treatment of antepartum IDA at our institution's obstetric antenatal testing centers (ATCs). These facilities are outpatient clinics designed primarily for the completion of routine obstetric ultrasonography and are not specialized infusion centers. This analysis was approved by the Institutional Review Board (IRB) and involved reviewing electronic medical records from January 2017 through June 2024. Our team received a list of all patients who received infusions at our institution's ATCs within the study period. All pregnant patients who received antenatal IV iron infusions at the ATC whose neonatal and maternal outcomes were available were included. There were no specific exclusion criteria aside from pregnant patients who received non‐IV iron infusions (for example, IV hydration for patients with hyperemesis gravidarum). Institutional referral criteria for IV iron included intolerance or poor response to oral iron (persistent low ferritin), Hgb < 10 g/dL at ≥34 weeks of gestation, or anticipated delivery prior to adequate oral iron repletion.

**FIGURE 1:**
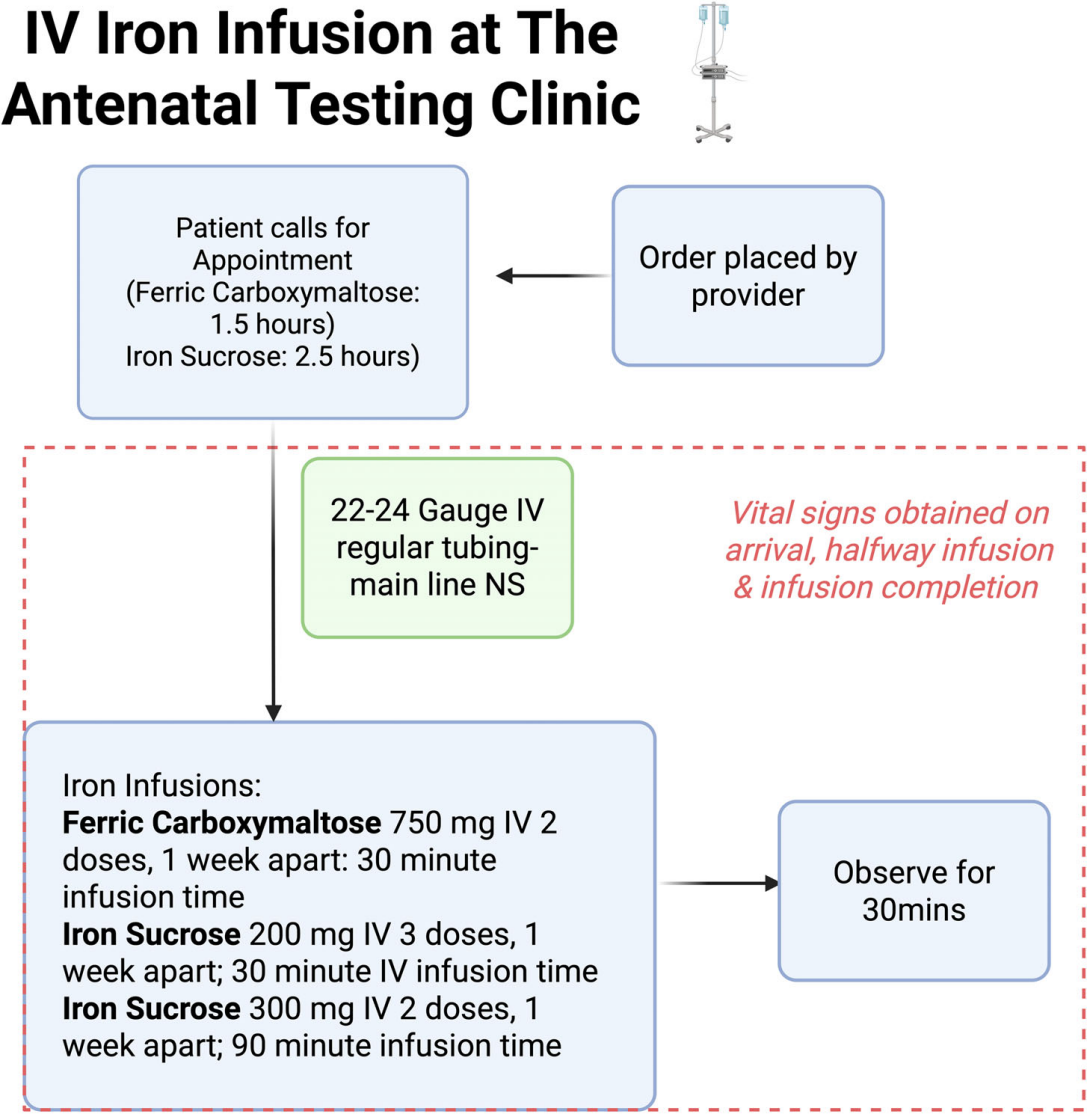
Illustrates the IV IRON Infusion protocol followed at the antenatal testing center.

In our clinic, the following iron solutions were available: Iron sucrose (Venofer), and ferric carboxymaltose (FCM; Injectafer). Iron sucrose was administered in dosages of 300 mg IV (two doses, one week apart, 90‐min infusion), or 200 mg IV (three doses, one week apart, 30‐min infusion). FCM was administered as 750 mg IV (two doses, one week apart, 30‐min infusion). The specific agent chosen and dose strategy were at the discretion of the ordering providers. Patient vital signs were obtained upon arrival, halfway through the infusion, and at completion, followed by a 30‐min observation period (see Figure 1 for protocol summary). No NSTs or fetal heart tones were performed throughout the visits.

Adverse reactions were managed based on severity. Mild reactions, including itching, flushing, urticaria, slight chest tightness, and nausea, were managed with infusion cessation for ≥15 min and vital sign monitoring. Moderate reactions, defined as transient cough, chest tightness, tachycardia, or hypotension, required the addition of IV saline bolus and possible corticosteroids to infusion cessation and monitoring. Severe reactions, including wheezing, stridor, cyanosis, and cardiac arrest, were managed with the above in addition to adrenaline, oxygen, and ACLS with transfer to the hospital for additional monitoring if clinically indicated. No patients received pre‐medication to prevent adverse reactions prior to infusion administration.

Data collection and abstraction were performed using RedCap. Demographic and medical history data included race, ethnicity, pregestational diabetes mellitus (DM), chronic or gestational hypertension (HTN), in vitro fertilization (IVF), advanced maternal age (AMA), hyperlipidemia, asthma, pre‐pregnancy anemia, autoimmune disorders, mental health disorders, cardiac conditions, obesity, thrombocytopenia, and hemoglobinopathy. Side effects, as well as maternal and neonatal outcomes, were also recorded.

The primary outcome of this study was to assess the safety and feasibility of outpatient IV iron infusion in an obstetric clinic setting, including the rate, severity, and management of adverse events (AEs: dizziness, headache, dyspepsia, urticaria, constipation, muscle cramps, flushing, hypotension, wheezing, stridor, cyanosis, cardiac arrest). Secondary outcomes included change in Hgb levels (difference between Hgb at delivery admission and pre‐infusion Hgb), the timing of the last IV iron infusion prior to delivery, maternal outcomes such as PPH, ICU admission, blood transfusions, and readmissions, and neonatal outcomes, including neonatal intensive care unit (NICU) admission, blood transfusions, and other adverse events.

Statistical analysis was performed using Shapiro‐Wilk or Skewness tests to check for normality and descriptive statistics of patient characteristics. Continuous data were presented as mean ± standard deviation (SD) or 95% confidence interval (CI), while categorical data were presented as *n* (%). Student t‐tests, Pearson's chi‐square, and Mann‐Whitney tests were used as appropriate. Secondary analysis was conducted based on the use of any oral iron supplementation post infusion, total iron dose, time of the last infusion from delivery, and the total number of IV iron infusions. Comparison by total IV iron dose (< 600 mg vs. ≥600 mg) was pre‐specified based on the common institutional target cumulative dose of ≥600 mg for effective iron repletion. Data were analyzed using STATA 18 (Stata Corp, College Station, TX, USA) and Prism 10.0 (GraphPad Software, San Diego, California, USA), with a *p*‐value < 0.05 denoting statistical significance.

## 3 RESULTS

### 3.1 Demographics and baseline characteristics

Out of 533 patients screened for intravenous iron administration, the study encompassed 417 eligible pregnant patients who underwent IV iron therapy at Inova ATCs. The racial distribution was 29.5% White or Caucasian, 14.9% Black or African American, 10.5% Asian, 39.3% Other, and 5.1% not reported. Regarding ethnicity, 43.3% were Hispanic, and 56.7% were non‐Hispanic. Insurance status was 53.4% private insurance, 43.5% Medicaid, and 3.1% uninsured (Table 1). The patient population included 1.2% DM, 4.8% chronic HTN, 1.7% hyperlipidemia, 8.6% asthma, 19.7% with reported prior history of anemia, 4.6% autoimmune disorders, 23.3% mental health disorders, 2.2% cardiac conditions, 22.3% obese, 2.4% thrombocytopenia, 2.6% hemoglobinopathy, and 28.3% with no significant medical history (Table 1).

**TABLE 1:** Baseline characteristics (*N* = 417). Data are presented as *n* (%: based on available data) or mean (SD) unless otherwise indicated.

**Demographics**		**Maternal medical history**	
Gravida	2.7 (1.7)	Pregestational diabetes	5 (1.2%)
Para: Living	1.2 (1.2)	Chronic hypertension	20 (4.8%)
Age	29.8 (6.9)	Asthma	36 (8.6%)
BMI	28.1 (6.4)	Mental health disorder	97 (23.3%)
White	121 (29.5%)	Obesity	93 (22.3%)
Black	61 (14.9%)	None	126 (30.1%)
Asian	43 (10.5%)	**Pregnancy complications**	
Other	161 (39.3%)	IVF	14 (3.3%)
**Ethnicity**		Gestational hypertension	16 (3.8%)
Hispanic	178 (43.3%)	Gestational diabetes	33 (7.9%)
Non‐Hispanic	234 (56.7%)	AMA	79 (18.9%)
		Multiple gestation	6 (1.4%)
		None	108 (25.9%)

### 3.2 IV Iron therapy types and dosages

Among the 417 patients, 87.3% received iron sucrose (Venofer), and 12.7% received ferric carboxymaltose (Injectafer); dosages varied; however, the majority (65.9%) received 600 mg total. Other common total dosages included 200 mg (2.2%), 300 mg (1.9%), 400 mg (4.3%), 900 mg (4.1%), 1000 mg (2.9%), 1200 mg (1.7%), 1500 mg (10.8%), and other (6.2%).

### 3.3 Impact on maternal hemoglobin

**FIGURE 2:**
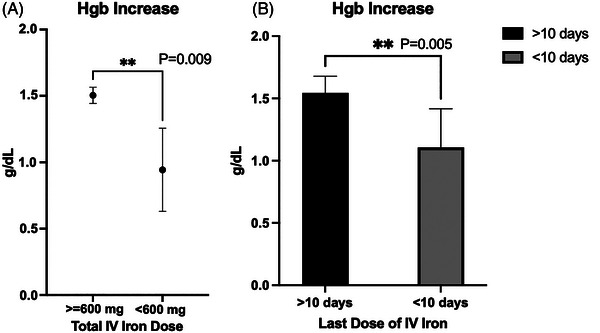
(A) Illustrates the rise in hemoglobin levels post‐infusion by total IV iron dose. Data is shown as mean and SEM. (B) Illustrates the rise in hemoglobin levels post‐infusion by the last infusion day. Data are shown as mean and SEM.

The mean gestational age at the first, second, and third infusions were 31.7 ± 4.6 weeks, 32.3 ± 4.4 weeks, and 33.0 ± 4.7 weeks, respectively. The baseline mean ferritin was 13.9 ± 26.4 ng/mL. The mean gestational age at delivery was 38.5 ± 1.6 weeks. There was a significant increase in Hgb from 9.7; 95%CI [9.6–9.8] g/dL at diagnosis to 11.2; 95%CI [11.1–11.3] g/dL at delivery (*p* < 0.0001). For those who received two or fewer doses of IV iron (*N* = 176), the median total iron dose was 600 mg (range 200–1500 mg) and the mean Hgb level increased from 10.1, 95%CI [10.0–10.3] g/dL at diagnosis to 11.5, 95%CI [11.4–11.7] g/dL at delivery, showing a mean change of 1.4, 95%CI [1.2–1.5] g/dL. In contrast, for those receiving three or more doses of IV iron (*N* = 178), the median total iron dose was 600 mg (range 500–2250 mg) and the mean Hgb level increased from 9.3, 95%CI [9.2–9.5] g/dL at diagnosis to 10.9, 95%CI [10.8–11.1] g/dL at delivery, showing a mean change of 1.6, 95%CI [1.4–1.8] g/dL. Hgb trends indicated similar improvement with three doses compared to two doses. When comparing total IV iron doses of less than 600 mg versus 600 mg or more, those receiving ≥600 mg (*N* = 341) had a significantly greater mean increase in Hgb (1.50 95%CI [1.13–1.62] g/dL) compared to those receiving < 600 mg (*N* = 37), who had a mean increase of 0.94, 95%CI [0.31–1.58] g/dL (*p* = 0.009) (Figure 2A); indicating that a higher total dose of IV iron was associated with a greater rise in Hgb levels.

### 3.4 Side effect profile

Out of the 417 patients, 11.5%, 95%CI [8.8%–14.9%] experienced any side effects (Table 2). The most common were dizziness (6.2%, 95%CI [4.3%–9.0%]), headache (1.9%, 95%CI [0.9–3.8]), and hypotension (1.7%, 95%CI [0.8%–3.5%]). Other side effects included dyspepsia (1.4%), urticaria (0.5%), constipation (0.2%), muscle cramps (0.5%), and flushing (0.2%) (Table 2). There were no cases of transaminitis, anaphylaxis, or infection. In only 0.5% of cases (*n* = 2), the infusion was terminated early due to moderate side effects. In one case, the infusion was paused due to abdominal pain, vital signs were taken, the patient was observed, and infusion was continued after in‐person evaluation by a nurse practitioner. No severe side effects were observed. No patients received any therapies for the observed side effects.

**TABLE 2:** Side effects observed (*N* = 417). Data are presented as *n* (%: based on available data) unless otherwise indicated.

Total incidence	11.5% 95%CI [8.8%–14.9%]
Mild reactions	
Dizziness	26 (6.2%)
Headache	8 (1.9%)
Dyspepsia	6 (1.4%)
Urticaria	2 (0.5%)
Constipation	1 (0.2%)
Muscle cramps	2 (0.5%)
Flushing	1 (0.2%)
Moderate reactions	
Hypotension	7 (1.7%)
Severe reactions	0 (0%)
Wheezing	
Stridor	
Cyanosis	
Cardiac arrest	

### 3.5 Maternal and neonatal outcomes

Maternal outcomes included a 4.8% incidence of PPH, 4.1% blood transfusion requirement, 0.6% diagnosed with hemorrhagic shock, 0.8% requiring ICU admission, and 3.3% readmitted within six weeks postpartum (Table 3). The composite maternal morbidity (PPH, shock index > 1 at time of delivery, blood transfusion, ICU admission, or readmission within six weeks postpartum) rate was 8.6%. Neonatal outcomes included a 7.4% NICU admission rate, 0.3% stillbirth rate, 3.5% of infants weighing less than 2500 g, 1.2% requiring transfusion, and 1.9% requiring intubation (Table 3). The composite neonatal morbidity rate (sepsis, meningitis, hypoglycemia, required surfactant, meconium aspiration, necrotizing enterocolitis, intraventricular hemorrhage, periventricular leukomalacia, or jaundice requiring phototherapy) was 18.2%.

**TABLE 3:** Secondary outcomes (*N* = 417). Data are presented as *n* (%: based on available data) or mean (SD) unless otherwise indicated.

Maternal and neonatal outcomes			
**Mode of delivery**		Stillbirth	1 (0.3%)
Vaginal	254 (61.0%)	Gestational age at birth (weeks)	38.5 (1.6)
Vaginal, Assisted	19 (4.6%)	Birth weight < 2500 g	14 (3.5%)
Cesarean (scheduled)	98 (23.5%)	APGAR (1 m)	7.9 (1.2)
Cesarean (unscheduled)	45 (10.8%)	APGAR (5 m)	8.8 (0.7)
Estimated blood loss (mL)	383.5 (271.0)	Neonatal hemoglobin	17.5 (3.3)
Required a uterotonic	123 (29.5%)	Neonatal hematocrit	48.6 (8.0)
Required > 1 uterotonic	50 (12.0%)	Neonate required transfusion?	4 (1%)
PPH (EBL > 1000 mL)	20 (4.8%)	Required surfactant	1 (0.2%)
Transfusion	17 (4.1%)	Hypoglycemia	55 (13.2%)
Units pRBC transfused	1 (0.9)	Meconium aspiration	7 (1.7%)
IV Iron postpartum	34 (8.9%)	Necrotizing enterocolitis	1 (0.2%)
ICU admission	3 (0.8%)	Intraventricular hemorrhage	2 (0.5%)
Readmission within 6 weeks postpartum	12 (3.3%)	Jaundice	22 (5.3%)
Maternal morbidity composite	36 (8.6%)	Intubation	8 (1.9%)
		NICU Admission	31 (7.4%)
		Neonatal morbidity composite	76 (18.2%)
Maternal composite—postpartum hemorrhage, diagnosis of hemorrhagic shock, ICU admission, readmission within 6 weeks postpartum		Neonatal composite—sepsis, meningitis, hypoglycemia, required surfactant, meconium aspiration, necrotizing enterocolitis, intraventricular hemorrhage, periventricular leukomalacia, jaundice requiring phototherapy	

### 3.6 Timing of intravenous iron therapy

When stratifying by the timing of the last IV iron infusion, we found that patients who received IV iron infusions more than 10 days before delivery experienced a significant increase in hemoglobin levels post‐infusion, with a mean rise of 1.5, 95%CI [1.4–1.7] g/dL compared to 1.1, 95%CI [0.8–1.4] g/dL in the less than 10 days group (*p* = 0.005) (Figure 2B). The maternal composite outcome (PPH, shock index > 1 at time of delivery, blood transfusion, ICU admission, or readmission within 6 weeks postpartum) rate was lower in the > 10 days group (6.9% 95%CI [4.4%–10.2%]) compared to the ≤10 days group (15.3% 95%CI [8.4%–24.7%]); unadjusted analysis demonstrated significantly increased odds of maternal composite morbidity for patients receiving IV iron ≤10 days prior to delivery (OR 2.43, 95% CI [1.17–5.02], *p* = 0.017). After adjusting for baseline hemoglobin, insurance status, maternal age, chronic hypertension, diabetes, obesity, and mental health disorder, this association was attenuated and no longer statistically significant (adjusted OR 2.00, 95% CI [0.92–4.32], *p* = 0.08). Lower baseline hemoglobin (OR 1.75, 95% CI [1.23–2.51], *p* = 0.002) and the presence of a mental health disorder (OR 2.27, 95% CI [1.05–4.90], *p* = 0.04) were independently associated with increased maternal morbidity. Composite neonatal outcomes showed no significant differences between the groups (17.5% 95%CI [13.5%–22.0%] vs. 21.2% 95%CI [13.1%–31.4%], *p* = 0.4).

### 3.7 Effect of oral iron supplementation

Among patients prescribed oral iron in addition to IV iron (*N* = 228), the mean hemoglobin (Hgb) level at diagnosis was 9.8 ± 1.0 g/dL, and it increased to 11.2 ± 1.3 g/dL at delivery admission. In comparison, those who did not receive oral iron supplementation during IV therapy (*N* = 150) had a mean Hgb at diagnosis of 9.7 ± 1.2 g/dL, which increased to 11.2 ± 1.1 g/dL at delivery admission. There was no significant difference between the two groups regarding hemoglobin increase from diagnosis to delivery admission (No PO Iron: 1.5; 95%CI [1.3–1.7] g/dL; PO Iron: 1.4; 95%CI [1.2–1.6] g/dL; P = 0.4). Of note, compliance with the prescribed regimen was not assessed.

## 4 DISCUSSION

This retrospective study demonstrates the feasibility and safety of outpatient IV iron therapy in treating IDA among pregnant patients. While multiple randomized trials have well established the efficacy of IV iron in improving hemoglobin, our study contributes novel data on the safe implementation of outpatient IV iron protocols in obstetric clinics without the need for inpatient monitoring or fetal surveillance.

Our findings suggest that IV iron therapy is well‐tolerated, with only 11.5% of patients experiencing side effects, the most common being dizziness (6.2%) and hypotension (1.7%). Notably, there were no cases of severe adverse reactions such as anaphylaxis or infection. These findings are consistent with the established safety profile of IV iron, which has been demonstrated in numerous studies to be both safe and effective for the rapid repletion of iron stores and hemoglobin levels [6, 10, 16, 17]. Compared to recent large‐scale safety data from Derman et al. [18], our overall adverse event rate of 11.5% falls between the reported rates for FCM (4.8%) and ferric derisomaltose (24.1%). Importantly, our study observed no severe adverse events, infusion‐related hospitalizations, or maternal deaths. At the same time, Derman et al. reported moderate‐to‐severe infusion reactions in up to 1.9% of patients and maternal serious adverse events (SAEs) in 4.8%–5.5%, including rare maternal mortality. These comparisons support the conclusion that outpatient IV iron administration in obstetric settings, when following a structured protocol, is both feasible and safe. None of our patients received pre‐treatment for the prevention of adverse reactions. This is consistent with the lack of current data supporting pre‐medication unless the patient is considered particularly high risk (multiple drug allergies, severe asthma, or prior infusion reaction) [14].

In our secondary analysis, patients who received oral iron supplementation in addition to the IV regimen showed only minimal additional improvement in hemoglobin levels compared to those who did not receive it, indicating that oral iron supplementation may not be necessary for patients already receiving IV iron therapy for anemia correction. Our study aligns with previous research demonstrating the advantages of IV iron therapy over oral supplementation, particularly in terms of absorption challenges and the rapid repletion of iron stores [8, 19]. The rapid increase in hemoglobin levels observed in our study underscores the importance of IV iron therapy in managing IDA during pregnancy, where timely correction of anemia is crucial for maternal and fetal health [3, 20, 21, 22].

The timing of the last IV iron dose appears to play a crucial role in optimizing maternal outcomes in antenatal IDA. Our analysis showed that administering the final IV iron dose more than 10 days before delivery was associated with a lower maternal composite morbidity rate in unadjusted analyses. After adjusting for baseline hemoglobin, maternal comorbidities, and socioeconomic factors, this association was attenuated and no longer statistically significant. These findings suggest that while earlier iron supplementation may contribute to improved maternal outcomes, other factors—particularly baseline hemoglobin and overall maternal health—also play a key role in determining risk. The latter two were independently associated with increased maternal morbidity. These results underscore the importance of early recognition and management of anemia in pregnancy and suggest that targeted interventions may be required for patients at highest risk of adverse maternal outcomes.

Although neonatal outcomes did not differ between the groups, the maternal benefits of earlier IV iron administration could inform future practice. Timely intervention may, therefore, be key to improving outcomes in pregnancies complicated by IDA.

Despite its benefits, IV iron therapy is typically more expensive in the short term and requires administration under supervision in specialized infusion centers or hospital settings. However, our study suggests that outpatient administration is feasible and effective. Notably, in our setting, no NSTs or fetal heart tones were performed, the visits were short, did not require additional resources, and were billed as outpatient treatments. This approach potentially reduces the burden on healthcare resources while maintaining high safety and efficacy standards. Of note, the majority of patients in our study were administered their total treatment dose in two to three sessions.

Recent recommendations for IV iron administration in the United States have shifted toward single‐infusion formulations such as low‐molecular‐weight iron dextran, ferumoxytol, FCM and ferric derisomaltose, which allow for rapid repletion of iron stores in one visit [15, 18, 23], thereby consolidating care to a single visit without increasing patient risks of toxicity. FCM is increasingly avoided due to its high incidence of hypophosphatemia and the logistical challenge of requiring two separate infusions [24, 25, 26]. Additionally, recent shortages of LMW iron dextran led to increased use of iron sucrose [27], which requires multiple infusions, emphasizing the need for accessible single‐dose alternatives. Consolidating care to a single infusion visit would further reduce burden on resources and patient. These trends reflect a growing emphasis on efficiency, safety, and patient adherence in IV iron therapy and the need for future studies to address these challenges.

Future studies should focus on cost analyses and the long‐term impact of IV iron therapy on maternal and neonatal outcomes and explore how to refine treatment timing and protocols to ensure optimal outcomes for both the mother and neonate. Evaluating hematologic parameters and maternal health‐related quality of life over extended periods, such as two years postpartum, could provide deeper insights into the enduring benefits of IV iron therapy. Additionally, investigating the impact of anemia and its treatment on long‐term maternal health and quality of life, beyond immediate hematologic improvements, is essential for comprehensive maternal care.

An important potential benefit of outpatient IV iron protocols is the opportunity to reduce disparities in anemia treatment access. Patients with limited financial resources, transportation barriers, or competing work and family obligations may be less able to attend prolonged hospital‐based or specialty infusion center visits. By delivering IV iron in outpatient obstetric clinics without the need for fetal monitoring or hospitalization, this model can expand access to timely and effective anemia treatment for a broader patient population. Such approaches may help address disparities in perinatal outcomes related to untreated anemia, particularly in underserved communities.

Strengths of this study include a large and diverse patient cohort, consistent outpatient clinical protocols across antenatal testing centers, and detailed tracking of both maternal and neonatal outcomes. The study also provides novel implementation data on outpatient IV iron administration without the use of fetal monitoring, which addresses a key barrier to broader clinical adoption. Limitations include the retrospective design, which may introduce unmeasured confounding, and the lack of systematic data collection on oral iron use prior to IV iron referral. Additionally, variability in total iron dose, driven in part by formulary restrictions and availability of iron sucrose as the primary formulation during much of the study period, may have led to lower cumulative dosing than the standard 1000 mg recommended in many guidelines; this reflects real‐world practice constraints and highlights the need for broader access to single‐infusion options.

Finally, while we adjusted for key maternal and clinical factors, residual confounding cannot be fully excluded, and the attenuation of the association between timing of last infusion and maternal morbidity in the adjusted model highlights the complexity of this relationship. Prospective studies will be needed to further refine optimal timing and dosing strategies and to confirm these findings in other practice settings.

In conclusion, our study demonstrates that outpatient IV iron therapy for IDA in pregnancy is safe, well‐tolerated, and feasible when administered without fetal monitoring or hospital‐based infusion. While earlier administration of IV iron was associated with lower maternal morbidity in unadjusted analyses, this association was attenuated after adjustment for baseline hemoglobin and maternal comorbidities, underscoring the importance of comprehensive anemia management. Our results provide practical guidance for the broader implementation of outpatient IV iron protocols, which have the potential to improve access to care and reduce healthcare resource utilization. Future prospective studies are needed to confirm these findings and to further refine optimal timing and dosing strategies for diverse obstetric populations.

## CONFLICT OF INTEREST STATEMENT

The authors declare no conflicts of interest.
